# Does the initial surface roughness of different CuNiTi wires affect the frictional resistance?

**DOI:** 10.1590/0103-6440202304912

**Published:** 2023-05-15

**Authors:** Bernardo Brenner Pentagna, Viviane Veroni Degan, Ana Paula Terossi de Godoi, Américo Bortolazzo Correr, Ana Rosa Costa Correr, Carolina Carmo de Menezes

**Affiliations:** 1 Department of Orthodontics, University Center of the Hermínio Ometto Foundation, Araras, SP, Brazil.; 2 Department of Restorative Dentistry, Dental Materials Division, Piracicaba Dental School, University of Campinas - UNICAMP, Piracicaba, SP, Brazil

**Keywords:** friction, orthodontic brackets, orthodontic wires

## Abstract

This study aimed to assess and correlate initial surface roughness and frictional resistance of rectangular CuNiTi wires inserted in different self-ligating brackets. The sample consisted of 40 bracket-wire sets (rectangular CuNiTi wires of 0.017" x 0.025" and passive self-ligating brackets) divided into four groups (n=10): metallic self-ligating bracket and metallic CuNiTi wire (G1); metallic self-ligating bracket and rhodium-coated CuNiTi wire (G2); esthetic self-ligating bracket and metallic wire (G3); esthetic self-ligating bracket and rhodium-coated CuNiTi wire (G4). The initial surface roughness of the wires was examined with a Surfcorder roughness meter, model SE1700. Later, frictional resistance was assessed in an Instron 4411 universal testing machine at a speed of 5 mm/min, in an aqueous medium at 35°C. Microscopic analyses of surface morphology were performed with scanning electron microscopy, using an LEO 1430, with magnifications of 1000X. Generalized linear models were applied, considering the 2 x 2 factorial (bracket type x wire type), at a 5% significance level. Regardless of bracket type, the groups with esthetic wires presented higher initial surface roughness than the groups with metallic wires (p<0.05). There was no significant difference between the different bracket-wire sets for frictional resistance and no significant correlation between frictional resistance and initial surface roughness in the environment studied. It is concluded that esthetic wires presented higher initial surface roughness but did not interfere with the frictional resistance between brackets and wires.

## Resumo

O objetivo deste estudo foi avaliar e correlacionar a rugosidade superficial inicial e a resistência a fricção dos fios CuNiTi retangulares inseridos em diferentes bráquetes autoligados. A amostra foi composta por 40 conjuntos bráquetes-fios (fios retangulares CuNiTi de 0.017” x 0.025” e braquetes autoligados passivos), divididos em 4 grupos (n=10): bráquete autoligado metálico e fio CuNiti metálico (G1); braquete autoligado metálico e fio CuNiti com revestimento de rhodium (G2); bráquete autoligado estético e fio metálico (G3); bráquete autoligado estético e fio CuNiti com revestimento de *rhodium* (G4). A rugosidade superficial inicial do fio foi examinada com um rugosímetro Surfcorder modelo SE1700. Posteriormente, a resistência a fricção foi avaliada em uma máquina de ensaios universal Instron 4411, a uma velocidade de 5mm/min em meio aquoso à 35^o^C. Análises microscópicas da morfologia de superfície foram realizadas por microscopia eletrônica de varredura, utilizando um LEO 1430, com ampliações de 1000X. Foram aplicados modelos lineares generalizados, considerando o fatorial 2 x 2 (tipo de bráquete x tipo de fio), com o nível de significância de 5%. Independentemente do tipo de bráquete, os grupos com fios estéticos apresentaram maior rugosidade superficial inicial que os grupos com fios metálicos (p<0,05). Não houve diferença significativa entre os diferentes conjuntos bráquetes-fios quanto a resistência à fricção e não houve correlação significativa entre a resistência a fricção e a rugosidade superficial inicial no ambiente estudado. Conclui-se que os fios estéticos apresentaram maior rugosidade superficial inicial porém não interferiram na resistência a fricção entre os braquetes e os fios.

## Introduction

The frictional resistance force between brackets and orthodontic wires during the sliding mechanics may complicate the effectiveness of dental movement due to the reduction of the force applied ^1^. In this context, bracket systems have been subjected to several changes, including the finishing of surfaces, bracket material, and different ligation methods ^2^, in an attempt to control the increase of frictional resistance.

Currently, there are several esthetic self-ligating brackets available in the market, which are composed of different materials such as polycarbonate and ceramics ^3^ ^,^ ^4^. The great advantage of these materials regarding metallic brackets is esthetics ^5^. When polycarbonate esthetic brackets are used along with esthetic wires, they may show different frictional resistance patterns due to both bracket composition and type of wire coating ^5^ ^,^ ^6^. Coated wires were developed with different coating materials, such as polymers or rhodium-coated wires ^7^, and the latter leaves a smoother surface and may reduce frictional resistance ^7^.

The literature reports that frictional resistance could be indirectly assessed with surface roughness ^8^, considering that frictional resistance would increase with the increase in the surface roughness of wires ^8^. However, some studies did not associate the increase in roughness with the increase in frictional resistance ^9^ ^,^ ^10^.

Surface roughness depends on wire type ^5^ ^,^ ^11^ ^,^ ^12^ and, among the different types, those made of CuNiTi have shown greater surface roughness than superelastic and round NiTi wires ^10^ ^,^ ^13^.

Surface roughness was also assessed in rectangular rhodium-coated NiTi wires, which showed rougher surfaces than non-coated wires ^14^ ^,^ ^15^. There are reports of higher frictional resistance of CuNiTi wires than rectangular TMA, NiTi, and stainless steel wires ^1^.

The literature reinforces the need for a deeper assessment with CuNiti wires ^1^, controlling the *in vitro* testing environment, as a dry environment may promote higher sliding and frictional resistance when assessing different orthodontic wires ^16^. The importance of controlling variables such as environment and temperature during *in vitro* tests becomes evident for assessing the correlation of surface roughness and frictional resistance of the different bracket-wire systems. Thus, this study aimed to assess and correlate the initial surface roughness and frictional resistance of rectangular CuNiTi wires in different self-ligating brackets, in an aqueous medium and a controlled temperature of 35°C. The null hypotheses of the study are the following: there is no difference between bracket types for frictional resistance; there is no difference between wire types for frictional resistance and initial surface roughness; there is no correlation between frictional resistance and initial surface roughness of orthodontic wires in the environment assessed.

## Materials and methods

### Sample production

The sample of this *in vitro* study consisted of two types of orthodontic wires: Twenty esthetic rhodium-coated CuNiTi wires and 20 conventional CuNiTi wires, both of the Orthometric brand (Marília, São Paulo, Brazil) with 0.017" x 0.025" of thickness. Two types of passive self-ligating brackets of upper right premolars were also tested: Twenty New e.volution esthetic self-ligating brackets of the New One orthodontics brand (Santos, São Paulo, Brazil) manufactured by Tecnident (São Carlos, São Paulo, Brazil) and 20 metallic self-ligating brackets of the Orthometric brand (Marília, São Paulo, Brazil). The sample resulted in 40 bracket-wire sets divided into four groups (n=10): metallic self-ligating bracket and metallic CuNiTi wire (G1); metallic self-ligating bracket and rhodium-coated CuNiTi wire (G2); esthetic self-ligating bracket and metallic wire (G3); esthetic self-ligating bracket and rhodium-coated CuNiTi wire (G4).

### Roughness assessment

The initial surface roughness of each wire type was examined with the Surfcorder roughness meter, model SE1700 (Kosaka Corp., Tokyo, Japan). This surface roughness meter was used with a diamond needle of 5 μm in diameter, at a constant speed of 0.5 mm/s. The cutoff value was 0.25 mm and the measurement length was 2.5 mm ^17^ (Albuquerque et al., 2017). Each wire type was aligned with the needle of the roughness meter. The initial surface roughness (Ra) of each orthodontic wire was read three times and the mean of the three readings was used for the statistical tests.

### Frictional resistance test

The method used to produce the support for the frictional test followed the methodology proposed in a previous study ^18^. Wire segments in different compositions (esthetic CuNiTi or conventional CuNiTi) of 5 cm were adapted to the support brackets. Both ends were folded with Nitinol wire folding pliers (DC Ortho, Tupã, SP, Brazil) to prevent the displacement of the wire from the slots during the friction test. These segments were fixed to the brackets with elastomeric ligatures (Morelli, Sorocaba, SP, Brazil), using a ligature applicator (Morelli, Sorocaba, SP, Brazil). The brackets tested were adapted to the wires in the open space of the support for sliding in the frictional test. A single operator performed all tests to better standardize the experiment.

To analyze frictional resistance, the acrylic plate was placed in an Instron 4411 universal testing machine (Canton, USA) within a glass recipient (200 x 190 mm in the base and 300 mm of height), with water at a temperature of 35°C.

**Figure 01:**
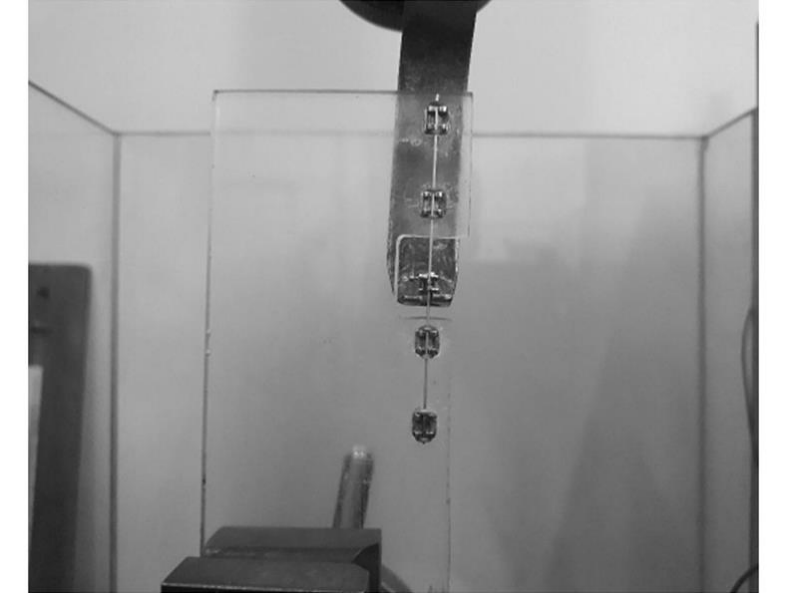
Schematic acrylic plate in position for testing.

For the bracket movement test, one end of the universal testing machine was used as a hook adapted with a lathe (Figure 01). Frictional resistance was analyzed at a speed of 5 mm/min and the peak and mean values were registered with the Bluehill 2.0 attached to the testing machine. The bracket was moved 5 mm in each wire and frictional resistance was assessed during the run. Three repetitions were performed for each set and their means were obtained in Newtons (N) as the measurement unit.

### Scanning electron microscopy

For analyzing surface morphology, each type of orthodontic wire was initially assessed. The upper end of each wire segment was fixed in a glass plate ^11^. A mark in the center of the wire was determined previously to standardize the reading. The central areas (0.025 inches) of the wires were observed with scanning electron microscopy (SEM), using an LEO 1430 (Carl Zeiss, Oberkochen, Germany). The images were obtained from secondary electrons, with magnifications of 1000X.

### Statistical analysis

The data obtained were studied initially with descriptive and exploratory analyses. The frictional resistance and roughness values did not meet the parametric assumptions; therefore, generalized linear models were applied, considering the 2 x 2 factorial (bracket type x wire type). A Pearson's correlation analysis was also performed between frictional resistance and surface roughness. The analyses were performed in the R software (Vienna, Austria), at a 5% significance level.

## Results

**Figure 02:**
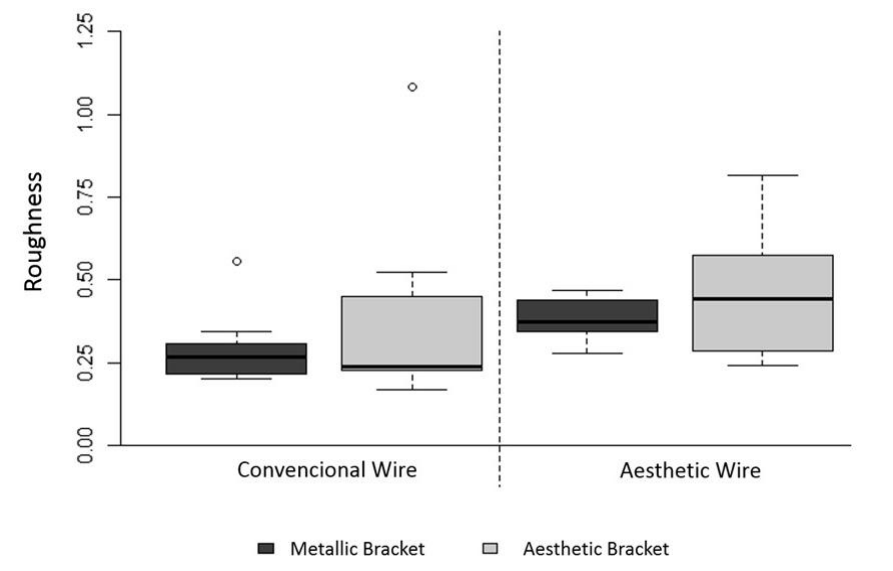
Box plot of roughness values for bracket and wire types.

Regardless of bracket type, the groups with esthetic wires presented higher initial surface roughness than the groups with metallic wires (p<0.05) (Figure 02 and Table 1). There was no significant difference between bracket and wire types for frictional resistance (p>0.05) (Table 1).

**Figure 03:**
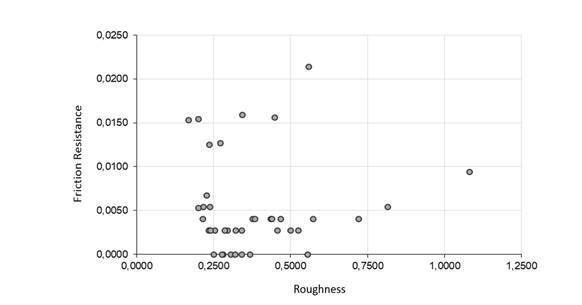
Scatter plot between resistance and roughness. r=0.0446 (p=0.8024).

There was no significant correlation between frictional resistance and initial surface roughness (p>0.05) (Figure 03).

**Table 1:** Mean (standard deviation) and median (minimum and maximum) of the frictional resistance (N) and initial surface roughness (μm) for the type of self-ligating bracket and wire.

Bracket	Resistance		Roughness	
	Wire type		Wire type	
	^3^ **Metallic** **Median (minimum and maximum)**	^4^ **Esthetic** **Median (minimum and maximum)**	^3^ **Metallic** **Median (minimum and maximum)**	^4^ **Esthetic** **Median (minimum and maximum)**
^1^Metallic	0.0027 (0.0000-0.0154)	0.0040 (0.0027-0.0159)	0.2650 (0.2003-0.5562)	0.3729 (0.2776-0.4372)
^2^Esthetic	0.0060 (0.0027-0.0156)	0.0040 (0.0027-0.0214)	0.2373 (0.1679-1.0820)	0.4424 (0.2392-0.8152)
p-values	p(bracket)=0.4560; p(wire)=0.5406; p(interaction)=0.9046		p(bracket)=0.1155; p(wire)=0.0444; p(interaction)=0.7747	

^1^Passive metallic self-ligating bracket; ^2^Passive completely esthetic self-ligating bracket; ^3^ Metallic CuNiTi of 017 x 025 inches; ^4^ Esthetic rhodium CuNiTi of 017 x 025 inches. Upper-case letters differ statistically in the row.

## Discussion

Some studies suggested that the surface roughness of brackets ^8^ and wires ^8^ ^,^ ^12^ would be responsible for higher or lower frictional resistance ^8^ ^,^ ^12^. The correlation analysis between them is of utmost importance to estimate the relationship of frictional resistance with surface roughness ^8^. Thus, this study aimed to assess and correlate initial surface roughness and frictional resistance of conventional and esthetic rectangular CuNiTi wires of 0.017 x 0.025 inches inserted in passive conventional self-ligating brackets, in an aqueous medium at a controlled temperature of 35°C.

The differentials found in the present study were the control of the *in vitro* testing environment, the frictional resistance test performed in an aqueous medium at a temperature of 35°C, and the choice of CuNiTi wires. The temperature of the aqueous medium was controlled according to the manufacturer's recommendations, and the value of 35°C was selected. Rhodium-coated esthetic wires were selected because they showed rougher surfaces than non-coated wires ^14^ ^,^ ^15^.

The results of this study did not show differences in frictional resistance between bracket types, which confirmed the first hypothesis. The literature has previously reported that frictional resistance force does not differ when comparing conventional stainless steel brackets with polycarbonate self-ligating brackets ^19^. Similar results were found for frictional resistance between conventional polycarbonate brackets and metallic brackets ^3^. This may have occurred because polycarbonate brackets are produced with small spheres of polymeric chains (macromolecules constituted by simple molecules) distributed homogeneously on the surface ^3^, not allowing a roughness increase. The literature reported that different frictional resistance forces are found when changing wire thickness and bracket-wire angle for passive self-ligating brackets ^20^. These characteristics were close in all the groups studied, which justifies the lack of difference between them.

The second hypothesis of the study was confirmed because there were no differences in frictional resistance. These results corroborate the study by ^7^, which reported that the frictional resistance forces of round and rectangular rhodium-coated NiTi wires could be equal to wires not coated with this material. Wires coated with rhodium, which is a noble and ductile white metal, are characterized by low frictional resistance, contributing to a higher force release during unloading ^5^. However, there are also reports of different frictional resistance between round and rectangular wires coated and not coated with rhodium ^21^ ^,^ ^22^. This difference was attributed to the increased wire-bracket angle in these studies, as wire diameters increased ^21^, which was maintained in the present study, and potentially attributed to the type of stainless steel wire ^22^ different from the ones used in this study.

Regardless of bracket type, the groups with esthetic wires presented higher initial surface roughness than the groups with metallic wires, which rejected the third hypothesis of the study. Directly comparing this result is not possible due to the absence of similar methodologies. The results by Rongo et al. (2014) showed higher roughness in rhodium-coated NiTi wires than in non-coated NiTi wires. This can be attributed to the different conditions of thermal treatment for the wires before and during the coating process with rhodium ^23^. The study by Trolić et al. (2017), found higher surface roughness for NiTi wires coated with rhodium. It is worth noting that these studies assessed the exposure of these wire types to saliva and probiotic supplement. Thus, it is suggested that the medium to which these wires were exposed may affect the surface roughness of these materials^24^. In the present study, initial surface roughness was assessed differently from the methodologies aforementioned.

There was no correlation between frictional resistance and initial surface roughness of CuNiTi wires. For different stainless steel wires, it is expected that frictional resistance tends to be higher for rougher surfaces ^25^. However, due to the difference in bracket material, the present study partially corroborates the results by Jaber et al. ^10^, which affirmed that a rougher surface of metallic NiTi and CuNiTi wires used with metallic self-ligating brackets does not relate to higher or lower frictional resistance. Therefore, it is suggested that the relationship of the type of wire composition assessed may affect the correlation between surface roughness and frictional resistance. The literature suggested that this relationship depends not only on the degree of surface roughness but also on roughness geometry, on the direction of roughness characteristics, and the relative hardness of both contact surfaces ^25^. The present study did not aim to assess different compositions of orthodontic wires, and new hypotheses can be tested.

**Figure 04:**
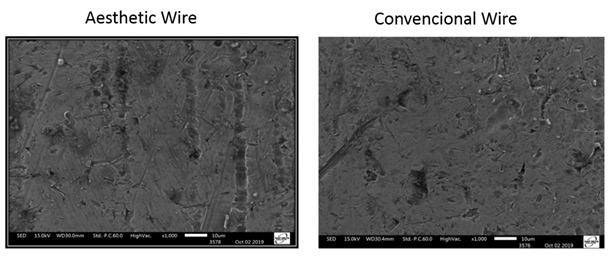
Scanning electron microscopy (SEM) images with magnifications of 1000X.

The assessment of surface smoothness and grooves of orthodontic wires with scanning electron microscopy found greater initial surface grooves in esthetic wires than in metallic ones (Figure 04). This qualitative assessment corroborates the surface roughness results of these different wires.

*In-vitro* studies are limited to simulating all existing clinical conditions involved in oral functions ^7^. Further studies are suggested to assess other conditions, simulating different clinical variables.

The present study verified the possibility of using rhodium-coated CuNiTi wires, which present satisfactory esthetics during the treatment and did not increase the frictional resistance. Thus, an esthetic and effective orthodontic treatment is possible. Despite the limitations of the *in vitro* study, it was concluded that rhodium-coated wires present higher surface roughness than metallic ones but both do not differ in frictional resistance behavior.
